# Is Information Asymmetry a Disruptive Factor in Food Consumer Behavior During the COVID Pandemic?

**DOI:** 10.3389/fnut.2022.912759

**Published:** 2022-06-30

**Authors:** Marian Socoliuc, Veronica Grosu, Marius-Sorin Ciubotariu, Simona-Maria Brînzaru, Cristina Gabriela Cosmulese

**Affiliations:** Department of Accounting, Auditing and Finance, “Ştefan cel Mare” University of Suceava, Suceava, Romania

**Keywords:** completive asymmetry, attributive asymmetry, food policies, sustainable products, business model

## Abstract

Today, food quality and safety, on the one hand, as well as increasing the level of information of consumers with direct implications on their food preferences, on the other hand, are highly debated topics in both national and international literature. The lack of consumers’ knowledge of information on food safety could make consumers purchase unsafe food. In the event of the existence of this kind of information, the consumption would be a safer one. Our research aimed to understand the means of adjusting the food offered to the request manifested by young and educated people and the impact of the information asymmetry on the consumers’ behavior. The main objectives of the study focus on exploring the nature of the informational asymmetry and the extent to which it usually affects the consumers’ perception and on identifying the prevalent socioeconomic factors that influence the consumers’ behavior regarding their perception of the quality of the food products and quantifying the impact of the information asymmetry on consumers’ behavior. In this study, a questionnaire survey among 529 young and educated people was used to design a cumulative analysis in order to allow the forecast based on a future trend of the food policies in relation to the change in the consumers’ behavior induced by the informational asymmetry. This analysis was segmented into impact sections that delineate the weight of the generating asymmetry factors from the weight of the factors with resistance to this phenomenon. The results of the survey led both to the identification of a quantification model of the information asymmetry that manifests itself within the relationship between the producer and the consumer and to the identification of a typology of informational asymmetry which manifests itself differently depending on the features of the food products. The study can be useful for those entities that want to identify the changes in the typology of consumption according to certain criteria in order to correlate their offer with the consumers’ requirements, as well as for the national or regional institutional bodies with a role in developing food policies adapted to these requirements.

## Introduction

The quality of food products in most European countries together with the illnesses that derive from a wrong consumption (i.e., malnutrition) pose a real threat to the health system. In order to combat this phenomenon, the EU has enacted numerous regulations and directives for ensuring food security. In this regard, the general requirements for food information in EU countries are described in Directive 2000/13/EC and in Regulation (EU) no. 1169/2011 (1). These documents aim to statute mandatory food information to be provided on food labels in order to substantiate consumer choices, starting from the fact that there is still an information gap between producers and consumers.

In recent years, we have witnessed a growing manifestation of obesity or overweight within the population in many industrialized economies and even in the developing economies. This is mostly a result of a lower quality of nutrition and a lack of the consumers’ adequate information in terms of the nutritional features and the quality of products they consume. A current factor that has significantly influenced consumer behavior has been the COVID-19 pandemic, which has affected people’s livelihoods and the global economy. In this context, information is essential to ensure the stability of final consumers in terms of their perception of the quality of food products, while also targeting spending decisions of them to achieve this quality (2, 3).

Our research aims to understand the means of adjusting the food offered to the request manifested by the target group (Romanian young people – students or university graduates) and the impact of the information asymmetry on the consumers’ behavior, using the questionnaire as a research method, for this purpose. The questions that make up the present questionnaire are part of the objectives that have been pursued through the Action Plan (4).

The following research objectives support the achievement of the present study’s aim: the first objective is to explore the nature of the information asymmetry and the extent to which it usually affects the consumer’s perception and behavior; the second objective is to examine the socioeconomic factors that determine the perception and behavior of consumers regarding the quality of food and its consumption; the third objective is to quantify the impact of the information asymmetry on consumers’ behavior; the fourth objective is to identify the causal relationship between this behavior and the food policies that have been developed by the national or regional institutional bodies at a given moment in time; and the fifth objective is the analysis of information asymmetry from the perspective of the consumer’s habitat.

Our findings contribute to the following aspects. First, we examined the existent information asymmetry between the producer and the consumer and its influence on the consumer’s behavior. Second, in this study, we used Gretl Statistical Program, version 2019 to develop an econometric model for designing a cumulative analysis. The aim was to allow the forecast based on a future trend of the food policies in relation to the change in the consumers’ behavior induced by the informational asymmetry. Finally, the results of the survey led both to the identification of a quantification model of the information asymmetry that manifests itself within the relationship between the producer and the consumer and to the identification of a typology of informational asymmetry which manifests itself differently depending on the features of the food products.

The rest of this study is divided as follows: section “Literature Review” provides a theoretical framework that helps develop assumptions that will be tested, as well as a review of the literature on the relationship between information asymmetry and consumers’ behavior. In section “Materials and Methods,” using a questionnaire survey applied to 549 young and educated people from Romania, we designed a cumulative analysis in order to allow the forecast based on a future trend of the food policies in relation to the change in the consumers’ behavior induced by the informational asymmetry. Section “Results and Discussions” reports the findings by presenting the conceptual model of the research and identifying a typology of informational asymmetry which manifests itself differently depending on the features of the food products. Finally, section “Conclusion” provides the limitations and suggestions for future research as well as the recommendations regarding the use of the proposed model on segments of consumers depending on the lack or the surplus of information in direct connection with the quality attributes of the information which are the basis for establishing food policies.

The study can be useful for those entities that want to identify the changes in the typology of consumption according to certain criteria in order to correlate their offer with the consumers’ requirements, as well as for the national or regional institutional bodies with a role in developing food policies adapted to these requirements.

## Literature Review

A number of publications have been developed on the topic of information asymmetry, but these mainly refer to the markets for financial services, insurance, used cars, or the labor market (5–7). The food market is rarely approached in terms of existing information asymmetry (8). We have recently witnessed an evolution of the modern consumer’s requirements as a result of the necessity of increased food safety due mainly to the restrictive conditions imposed by the global COVID-19 pandemic (9). In terms of food safety, it has become an indispensable requirement. However, it seems that consumers consider it a priority mainly in the case of those issues that increase the perceived risk regarding certain aspects of production technologies [i.e., genetically modified products (GMOs), irradiation, etc.] (10, 11). These changes were the result of the transformations and dynamics that took place in the “reflexive modernization” and the “risk society” (12). Within these aspects, the demand for biological, quality products represents the consumer’s attempt to surpass the information asymmetries (5) in a broader sense with respect to the consumed food in order to construct their own identity based on the way of living.

The elimination of the condition of perfect information regarding the quality of the offered products generates certain issues that consumers face such as the moral hazard – when the sellers choose the quality of the product, that is when the quality is endogenous and the adverse selection – when goods of different and unperceived qualities are offered to buyers, that is when the quality is exogenous. The most well-known definition of the concept of information asymmetry that exists in the economic literature belongs to Stigler (13) who considers it as “looking for information by the consumer regarding the qualitative features of a product in relation to the cost-benefit analysis.”

The problem of information involves a double form of manifestation: incompleteness and asymmetry. Incompleteness refers to the fact that not everyone owns all the relevant information. Yet, asymmetry refers to the fact that the information is not evenly distributed and that some have confidential information. This means that it is inaccessible to others. Both issues are relevant because the information has a cost, namely the cost of searching and collecting it, as well as the cost of missing information (14). Less information can lead to the loss of individual and collective wellbeing that can have negative external effects. The cost and the possibility of gathering information on the quality of goods facilitate the distinction among search goods, experience goods, and credence goods (15, 16).

Technically speaking, there is negative outsourcing and therefore, there is a market failure associated with the overconsumption of food (17), especially in the current environment, which is affected by the COVID-19 pandemic when risk management for the general food system involves establishing fair market rules and ensuring an open exchange of information in food supply chains (18). In fact, the authors analyzed the way the nutritional policies have evolved in both the developed and the developing economies. They explore all the factors that explain nowadays those trends in nutrition, health, and maintaining weight and the results of the cost-benefit analysis.

Another factor that has affected the food market is the consumer panic caused by the health pandemic, which has brought impacts on food safety in several ways, such as on the supply and demand of food or changes in consumer preferences (19). The authors show that income was the main reference in the decisions to consume food products and the importance of animal welfare, environmental sustainability, information on the origin and manufacture of food, the appearance of food, correct payment to the producer, and packaging of food were given a lower reference. Therefore, in times of crisis, consumer behavior changes in an attempt to adapt to the environment, and reducing information asymmetry would significantly contribute to the consumer’s stability and, at the same, to ensuring the resilience of the sector during this crisis.

The distance between the producer and the consumer is essentially informational (20). Starting from the dynamics of the relations existing between these parts, we can identify the fact that certain characteristics of the product offered by the manufacturer are transmitted to the consumer based on their own communication capabilities. These characteristics are perceived by consumers as attributes of the product and are based on their individual abilities, values, and beliefs. Those attributes to which the consumer gives value represent the determining factors of the purchase decision. Often the consumer is not able to interpret the information needed to evaluate the characteristics of the purchased and consumed goods. Therefore, the information about these characteristics cannot meet the conditions for good information. This situation highlights the fact that, in the food market, in the absence of regulations, a high degree of informational asymmetry is generated, which results in a faulty functioning of the markets.

**FIGURE 1:**
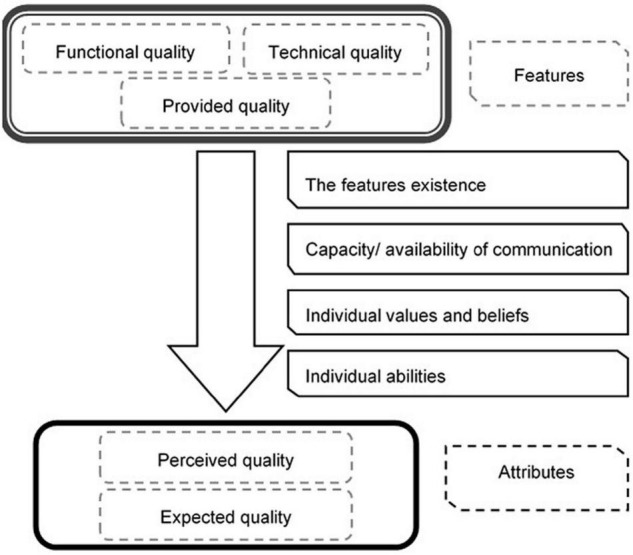
The relation between quality in terms of food safety, attributes, and features. Source: 20.

We present the abovementioned aspects in Figure 1 as follows:

Food quality is a topic of ongoing relevance. It is of great interest both to all the individuals regardless of age, sex, level of education, or region of origin and to the producers, as they must always take into account the consumers’ behavioral requirements and changes. In this context, the information asymmetry phenomenon has a double impact, both from the point of view of the demand for food and the supply of food. The management of this phenomenon by taking the consumers into consideration can have a significant economic, financial, and social effect on the producers and, most importantly, on the sustainability of their businesses (21).

The information asymmetry refers to many aspects. Some authors have discussed the relationship between intangible assets, asymmetric information, relationship banking, financing costs of SMEs, and the usefulness of quarterly reports for investors in times of uncertainty such as the COVID-19 pandemic (22, 23). The literature most often refers to the asymmetry in connection to the features of the product. It is no doubt a core area where we can talk about the asymmetry of information that manifests between the food manufacturer and the buyer (24). The role of both information asymmetry and product quality has been explored extensively in the specialty literature. It serves as the basis for the below models (see Table 1).

**TABLE 1:** The synthesis of the main impact studies on the researched field.

Author	Aim	Results/Conclusion	Impact
Zhai and Han (59)	To examine whether the certificate authority’s inspection information sharing policy (IIS policy) can improve food quality in online commerce.	The results suggest that producers tend to offer high-quality food, while both the online platform and food producers can earn more profits by adjusting the selling price and commissions according to IIS policy.	A high impact study because, during the COVID-19 pandemic, e-commerce in the area of food products has increased considerably but exacerbates the information asymmetry, which generates a market full of low-priced and poor-quality food. It is a phenomenon that must be followed carefully.
Bronnmann et al. (60)	To design discrete-choice experiments for measuring the relative importance of the motivations for choosing eco-labeled products in order to reduce the information asymmetry between producers and consumers.	The result shows that choice probability increases if the product carries an ecolabel (a percentage of 63% choice probability to consumer demand for sustainable products), but the magnitude of this effect depends on the information provided about the sustainability of the product.	An average impact is due to the fact that this study emphasizes a primary tool in providing information between producer and consumer, namely eco-labels of products.
Rezitis and Tsionas (32)	This study focuses on “a multivariate panel error correction model (PVECM) to investigate asymmetric price transmission among the farm, processor, and retail segments of the European food supply chain” for the 2005–2016 period.	The results indicate that, in both the long- and short-run, retail prices respond more strongly to processor price increases than decreases and the same occurs for processor prices due to farm price changes. Thus, the findings demonstrate the presence of “positive asymmetric price transmission in the European food supply chain.”	High impact due to the fact that the study supports the general findings of the existing literature, namely that the food price pass-through varies greatly across the product category and across counties as well as that the pass-through to producer prices is greater than to the consumer prices.
Galati et al. (11)	Identifies the main factors affecting the consumers’ interest in receiving information on food irradiation technology.	Findings revealed that 89.2% of the Italian consumers are interested in receiving information on the treatment of foods with ionizing radiation aimed at raising product safety.	High impact due to the fact that the study underlined that a concern of misinformation manifests among the Italian consumers regarding the irradiation technology.
Brach et al. (61)	To investigate the potential moderating effect of the perceived credibility of third-party certification labels (TPCL) on both perceived risk and purchase intentions.	Sustainable products are characterized by credence qualities that are associated with increased perceptions of risk, which negatively influence the consumers’ purchase intentions. TPCL on sustainable products provides brand-like information cues that reduce the perceived risk of the sustainable products. Consumers must perceive TPCL as credible for them in order to reduce the consumers’ risk perceptions.	It is an impact study as it addresses those companies that allocate resources to sustainable products and those legislators that adopt the policy in this area.
Plank and Teichmann (62)	To suggest a new product label, build a facts panel on Corporate Social and Environmental Behavior (CSEB Facts Panel), and test its effects on the consumers’ response.	Results showed that, when consumers face a trade-off between social responsibility and environmental responsibility, social responsibility is more important to consumers and individual difference variables, such as social consciousness and environmental consciousness, can influence consumers’ responses to the CSEB Facts Panel.	Average impact of the study due to its experimental nature; this research only tested three specific facts for corporate social behavior and corporate environmental behavior each.
Le and Nguyen (31)	To study the way the market for safe vegetables works in Vietnam and how it responds to the government’s inspection by suggesting a theoretical model that depicts a shop owner’s behavior within the market for safe vegetables under informational asymmetry that manifests between the sellers and the buyers.	Results show that low credibility leads to limited efficiency of the inspection activities. Having a third party for quality inspection improves the situation. Not only the mix ratio but also the total of truly safe vegetables consumed are increased alongside the likelihood of inspection.	The average impact is based on the fact that the conclusion from this study can also be applied to other credence goods.
Palma et al. (33)	To analyze whether asymmetry in information affects consumer preferences and willingness to pay for ambiguous claims using the native attribute.	The results showed that consumers preferred native varieties. Furthermore, tastes and preferences for all product attributes were heterogeneous; heterogeneity in preference for the native attribute was only significant at the 10% level.	The average impact is based on the fact that the results provide additional evidence that the consumers might be misled by their perceptions of “native” products with incomplete or ambiguous information.
Nestorowicz (24)	To examine the areas affected by the asymmetry of knowledge and information between producers and consumers in the food market.	The results show that if information asymmetry is reduced, it may be said that it is a socially responsible communication; otherwise, we cannot talk about CSR.	The average impact is based on the fact that the study offers clues about the actions of the manufacturer which could reduce or deepen the asymmetry.

*Source: Author’s compilation.*

From the analysis of the specialized literature, we observed that the phenomenon of informational asymmetry manifested in the food market is addressed in a few specialized studies. Information asymmetry is a complex and broad field of research, as more studies are needed to design and evaluate food policies. Although providing more information could lead to a reduction in information asymmetry, there is evidence in the literature that, under certain circumstances, consumers would be affected by the information avalanche (25, 26). Some studies have found that consumers’ attitudes and behaviors toward production practices and locations may vary even depending on the nationality of the consumer, that product labeling works differently depending on the type of product and the consumers’ reaction to information is temporary, and that education has no effect on students’ risk perception (27, 28). However, in the context of certain factors affecting the global economy such as the current COVID-19 pandemic, age, and level of education are key elements in changing consumer behavior, especially for consumers’ attitudes toward sustainable food choices (29).

The starting point in our scientific approach was the research elaborated by Palma (30) in which the author suggests that future research should analyze whether policies such as labeling effectively increase consumer welfare and affect consumer preferences. In this respect, in order to provide a clearer image of the concepts, we resorted to the structuring of the meta-analysis on the related phenomena based on the technique of dissociation phenomena; the existing correlation between the informational asymmetry between the sellers and the buyers (31); the information asymmetry and the consumers’ interest (11) in the asymmetric price transmission within the food supply chain (32); the relationship between the consumers’ preferences, their willingness to pay for ambiguous claims, and the asymmetry information (33); and the information asymmetry and the social responsibility (24).

As far as the information asymmetry between the consumers and the producers is concerned, the following situations can be identified: the presence of a high level of information asymmetry, the existence of the generic information, and the rarest case in which information and specific knowledge are held. We believe that, by providing correct and clear information on the demand for ecological products, the chances of future development for the marketing of these products are visibly increasing.

## Materials and Methods

Most of the studies on informational asymmetry and its influence on consumer behavior regarding food choices have been conducted predominantly among adult consumers and less among young and educated consumers. In this sense, we consider it of real interest to study the behavior of young consumers and especially the educated ones. They represent an interesting consumer segment to explore consumers’ perception of food, given that they have a growing purchasing power and that the presence of children and adolescents in a family influences the level and the structure of spending and consumption in the household (34–36). The reason why we decided to select this target group was due to attempts to identify to what extent the educated people (more numerous than those with an elementary education) know, realize, and follow a diet with quality/healthy products and to what extent they read and study the labels that certify the food quality and safety.

The research is needed to diagnose the consumption trends in this segment of the market, to allow the creation of a sustainable market offer adapted to this group of consumers, and to study the impact of the informational asymmetry on the change in consumption behavior. We consider that this desire together with the complexity of the problems related to sustainable consumption fully justifies the opportunity of the study carried out in this study. In our study, we found the answer to the following research questions: (1) Does information asymmetry affect consumer perception and behavior, and to what extent? (2) What are the determining factors on consumers’ perception and behavior regarding the quality of the products? (3) Is there a causal relationship between this behavior and the food policies developed by national and international bodies? (4) Is there a causal relationship between the consumer’s habitat and the information asymmetry?

Knowing the changes in consumer behavior influenced by information asymmetry, as a disruptive factor in food policy and in consumer decision making, which is the result of this survey, will allow the adaptation of food policies to the needs of consumers.

**FIGURE 2:**
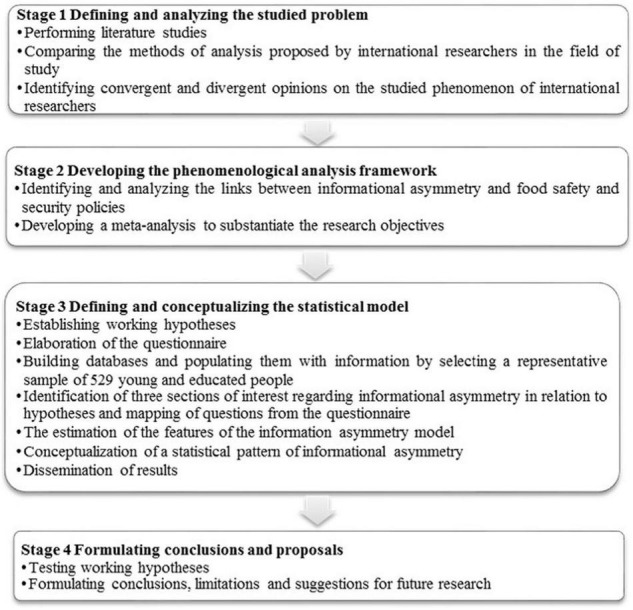
Mapping of the research. Source: Author’s compilation.

**FIGURE 3:**
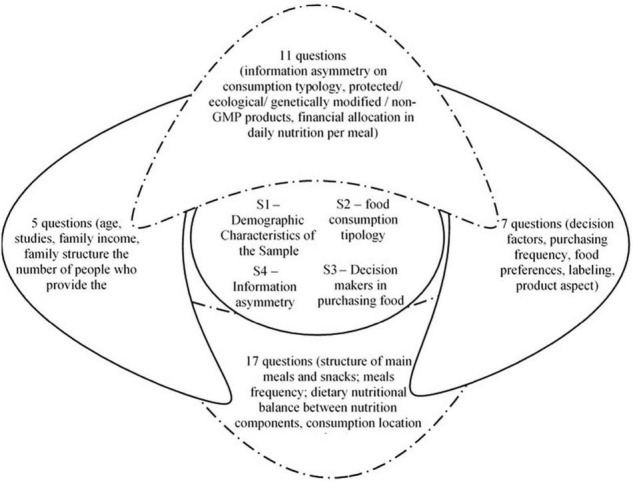
The structure of the questionnaire (*S-sections). Source: Author’s compilation.

The mapping of the research aims at reaching each point and finalizing all the four stages is shown in Figure 2:

To achieve the research objectives, we used the prospective survey in the form of a questionnaire as a research method. In this regard, we developed a questionnaire composed of 40 questions structured in four sections of impact, as can be seen in Figure 3. In the construction of the sample used in the present research, the authors applied certain inclusion and exclusion criteria:

1. The inclusion criteria: the sampling relies on the principle of considering the educated people as part of a period of vocational training, as those who attend university or post-graduate courses, and as those who have access to information channels;
2. The exclusion criteria: those individuals who declined the answers to all the questions from the survey.

The questionnaire survey was applied to 549 young and educated people in 2019 (the sample was made of Romanians). Out of 549 completed questionnaires, only 529 could be interpreted, because 20 of them were excluded due to the fact that the respondents did not provide answers to at least half of the number of questions in the questionnaire. This sample is made up of students, masters, doctoral students, and graduates from universities located in the N-E region of Romania. In this respect, the answers were analyzed and interpreted depending on the level of education – in our case the target group is made up solely of people with university degrees or attending a university at present, on the social and economic conditions or the workplace. The survey included students from all three cycles of higher education and graduates with their ages ranging mainly between 18 and 35 years, studying both in the fundamental field of exact sciences and in the field of fundamental social sciences. The questionnaire was conducted using Google Drive electronic platform. The data collected from responders through the electronic platform was centralized through the Microsoft Office Excel program. Statistical processing techniques were applied to this database, consisting of scaling the answers on relevant intervals for the questioned items; transforming qualitative data into quantitative data; measuring modal distances between intervals through quartile intervals and standard deviations; and weighting the information obtained in order to quantify the relevance of the results of the questionnaire and their graphical interpretation. The database was analyzed on certain criteria depending on the answer options to the questions of the questionnaire and quantified on the basis of the specific consistency of the answers in relative representation data of the phenomena suggested to the study based on the questionnaire. The relative data interpretation was done through the weighting method between the minimum and maximum barriers. Based on the statistical software Gretl, version 2019, we generated a model of information asymmetry.

To understand the means of adjusting the food offered to the request manifested by the target group and the impact of the information asymmetry on the consumers’ behavior, five hypotheses were advanced:

*H1:* The information asymmetry is a factor that manifests itself in the relationship of direct proportionality with the level of education of the population and with the financial capacity of sustaining the global consumption per household.

*H2:* The informational asymmetry occurs when the need for the consumption of quality health products exceeds the financial resources to cover those needs.

*H3:* The informational asymmetry is the main factor generating the change in the food policy in relation directly proportional to the typology of the consumption of the young and educated population.

*H4:* The informational asymmetry is influenced by the migration of the young and educated consumers and by the traditional local customs.

*H5:* Information asymmetry has a direct impact on the behavior of young and educated consumers, influencing both the purchase decision and the structure of the budget allocated to food consumption. In order to assess the impact of the information asymmetry on the consumers’ behavior, we have drawn up a questionnaire (see Figure 3).

The questionnaire has been structured into four sections in order to meet the objective suggested by the research, namely to determine whether the information asymmetry affects the consumers’ behavior by pointing out the impact on the purchasing decision and on the outline of a personalized diet.

The hypotheses outlined above will be encoded with Hi, with *i* = $\bar{1,5}$. They correspond to the objectives established by the study that was previously defined. The questions in the questionnaire will be grouped into three sections, namely characteristics, attributes, and causality. The sections will be compiled in with the mapping of the questions *Q* = $\bar{1,40}$. In the following section, we have reported the results of a survey.

## Results and Discussion

For a better presentation of the study results and their correlation with the set hypotheses, in the following, we proceeded to group them into sections. The grouping procedure followed the same logical reasoning used in the structure of the questions in the questionnaire according to Figure 3.

### Demographic Characteristics of the Respondents

The questionnaire was completed by 549 people, most respondents were aged 24 or younger, with the most representative age group between 20 and 25 years (46.36%). Students younger than 18 years old were not admitted to this study. About 20 incomplete questionnaires were excluded. Table 2 presents the characteristics of those who successfully completed the survey questionnaire (*n* = 529). The profiles of the respondents were differentiated by age, studies, family income, family structure, and the number of people who provided the average income family.

**TABLE 2:** The structure of the food consumption of families with one or more members contributing to the family income.

Working members	Milk and dairy products (%)	Eggs and other products of animal origin (%)	Cereals and bakery products (%)	Tea and coffee or other hot drinks (%)	According to personal preferences (%)
All	15.48	23.86	17.30	34.43	8.93
1 member	16.49	26.60	14.89	31.91	10.11
2 members	14.84	22.26	18.73	36.40	7.77
3 members	18.37	18.37	20.41	34.69	8.16
More than 3 members	10.34	31.03	13.79	31.03	13.79

With regard to the level of their education, the majority of the responders (i.e., 369 individuals) attended bachelor’s studies. A total of 129 individuals are engaged in long-term higher education (i.e., master’s degree). From the point of view of doctoral studies, only 1% of the people who were questioned attend this type of education.

The labor income of the people questioned is a significantly reduced one in the sense that 48% of responders earn an average monthly salary of up to 300 euros, which is the equivalent of the net minimum wage whereas 33% of the people questioned earn an average salary up to the level of the average income (i.e., 1,000 euros maximum). A number of 93 respondents (16.94%) earn a reasonable average monthly salary of over 1,000 euros.

From the point of view of the family structure, the majority of the respondents (69.03%) declared to be a part of the traditional family with many children who were dependents. A total number of 26 people declared that they are a part of a single-parent family whereas 72 respondents (13.11%) declared that the family they belong to has no children or they are the only child.

The number of individuals that earn the average income is mainly a part of the sampled 2-person group (51.55%) – a significant statistical percentage for the sampled population (34.24%) – declared that only a single member earns the family income. This proves the low value of the average family income below 300 euros for the majority of the people questioned (49.18%). Our study confirms the results obtained in previous studies by other researchers (19, 37–39), according to which the frequency of purchase is directly influenced by respondents’ incomes so that an increase in income denotes a high percentage of consumers who buy food.

The structure of the information within the questionnaire was centralized. This thing led to the segmentation picture as shown in Table 3.

**TABLE 3:** The structure of the respondents’ options on features related to age, degree of education, and other family features.

1. Age		2. Studies	
18–20	33.33%	Student	67.21%
20–25	47.36%	Short-term higher education	8.20%
25–30	7.83%	Long-term higher education	23.50%
Over 30	11.48%	Ph.D.	1.09%
**3. Average family income**		**4. Family composition**	
>300 euro	22.22%	1 member	13.11%
300 euro	26.96%	2 members	13.11%
300–1,000 euro	33.88%	3 members	69.03%
Over 1,000 euro	16.94%	More than 3 members	4.74%
**5. Working members**			
1 member		34.24%	
2 members		51.55%	
3 members		8.93%	
More than 3 members		5.28%	

Another aspect analyzed in this section was the structure of the food consumption of families with one or more members who contribute to the family income (see Table 2).

From the above data, it can be observed that a higher allocation from the budget allocated to the food consumption of milk and dairy products and cereals and bakery products appears in the case of families with three members. This aspect suggests that the family also has in its component a young person, which, in our opinion, changes the level and structure of the budget of food consumption in the household. The analysis also showed that families with more than three members allocate more financial resources for the consumption of eggs and other products of animal origin (31.03%). A final aspect of the analysis was the fact that the largest spending from the budget on tea and coffee or other hot drinks (36.40%) is by families with two members, which confirms that they spend more time socializing in different locations where these products are marketed.

### Food Consumption Typology

The consumption preference (i.e., the second section) regarding the place where meals are being served is mainly represented by the respondents’ households (72.13%), and a significant number (15.3%) are made of the student population who use the university cafeteria as a place for having a meal. This option corresponds to those individuals that do not reside in that specific city and are usually accommodated in student dormitories. For them, the option of using the cafeteria represents an indicator regarding the quality-price ratio. Usually, the first meal of the day, the breakfast, mainly consists of tea (34.43%), eggs and other products of animal origin (23.86%), and bakery products (17.3%). Serving dairy products is preferred by 15.48%. The last place is occupied by those who prefer personalized menus based on preferences (8.93%). Lunch-time during regular days consists of chicken (36.98%) and potatoes, rice, or bakery products (32.9%). A relatively rare habit in the consumers’ options is given by pork consumption (9.47%) and meals that mainly consist of fruits and vegetables (7.47%). Unlike regular days, during the weekend, the respondents admitted to doubling their pork consumption (18.58%) and a 40% cut down on their potato, rice, and bakery product consumption. Both the fruits and vegetables consumption (8.93%) and the chicken consumption remain constant. Neither on regular days nor during the weekends have they preferred sweets at lunch (a little over 5%). These aspects are shown in Table 4.

**TABLE 4:** The structure of the change in pork consumption depends on the festive or ordinary moment of the day and (depending on) the members that contribute to the family income.

Pork meat	Lunch (%)	Holiday lunch (%)	Dinner (%)	Week-end dinner (%)	Holiday dinner (%)
All	9.47	35.15	5.83	10.93	20.04
1 member	10.64	31.91	5.85	9.57	18.09
2 members	9.54	38.52	6.36	12.01	22.61
3 members	8.16	32.65	4.08	8.16	12.24
More than 3 members	3.45	27.59	3.45	13.79	20.69

During holidays, pork consumption reaches 35.15% of the consumption preferences of the interviewed population, while poultry is cut down to 23.5%, compared to 36.98% during regular days. A significant reduction in potato, rice, and bakery product consumption registers an increase during the holidays compared to the regular days. Thus, if on regular days the consumption of such products reaches 32.97%, during the holidays the consumption decreases by 5.65%.

There is a significant change in the consumption of beef products too. Since there is a 2.73% consumption during the regular days, it registers a 10.75% increase during the holidays. The same trend takes place for sweets whose consumption increases during the weekend and during the holidays by 8.2% of the population’s preferences.

Fish consumption was observed to decrease among respondents to only 2.19% during regular days and to 9.47% during the holidays. An interesting observation mentioned by the study of the consumers’ options depending on the festive significance of lunches is the fact that those individuals who choose to have personalized menus (i.e., persons being on a diet, or persons submitted to diets) do not change their consumption options depending on the festive significance of the moment. The percentage of respondents in this situation is 4.55% of the total questioned population.

As far as dinner is concerned, the majority of respondents consider it as their the second main meal of the day. As shown in Table 5, on regular days, it consists mainly of fruits and vegetables (36.25%), potatoes, rice, or bakery products (26.05%), and chicken (18.76%).

**TABLE 5:** The structure of the change in the chicken consumption depends on the festive or ordinary moment of the day and (depending on) the members that contribute to the family income.

Chicken meat	Lunch (%)	Week-end lunch (%)	Holliday lunch (%)	Dinner (%)	Week-end dinner (%)	Holiday dinner (%)
All	36.98	33.88	23.50	18.76	19.13	20.58
1 member	33.51	27.13	25.53	17.55	20.21	23.40
2 members	38.16	36.04	23.32	20.85	19.08	18.73
3 members	42.86	46.94	16.33	12.24	16.33	18.37
More than 3 members	37.93	34.48	24.14	17.24	17.24	24.14

The rest of the consumers’ preferences regarding sweets, pork, beef, and fish consumption are placed below 5%, not influencing significantly the quality of dinner. Changing preferences during the weekend was seen to be influenced, especially by the decrease in the consumption of fruits and vegetables for dinner and the increase of pork consumption (by doubling this consumption from 5.83 to 10.93%). One can also see structural changes such as a quantitative increase in fish consumption (by tripling the consumption from 2.73 to 9.47%). The sweet consumption changes from 3.83 to 4.92%, and the poultry consumption slightly increases from 18.76 to 19.13%. With regards to dinner during holidays, it mainly consists of poultry consumption (20.51%), pork (20.04%), fish (10.56%), fruits and vegetables (15.66%), potatoes, rice, and bakery products (10.56%), and sweets (19.11%).

The questionnaire disseminated on the basis of the significant weightings shows that, out of the three daily main meals, dinner time during the holidays has the most diversified structure in the sense of a balanced consumption of all nutritional elements whereas lunch is the richest in nutrients, and breakfast is a meal considered by most respondents as frugal and slightly dispensable. With regards to festive moments, one can notice that, during holidays, the meat consumption increases, especially the pork consumption whereas the amino acid (i.e., starch) consumption decreases. Fruits and vegetables do not occupy a preferential place in the consumption typology of the respondents. This shows that in spite of the fact that the level of culture is high, the structure of the food pyramid (40) is not accepted for reasons that have more to do with the food tradition than with the food philosophy of the respondents.

**FIGURE 4:**
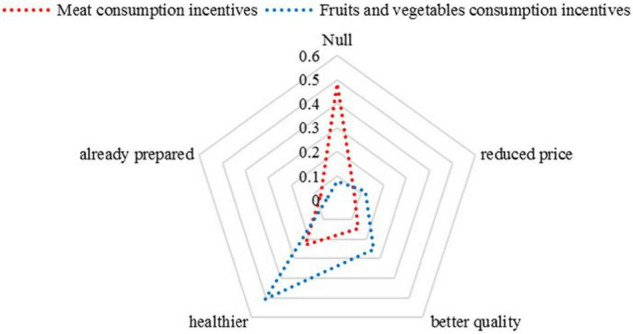
Differences in options regarding the consumption and motivation in consumption of meat products and/or fruits and vegetables.

About 72.68% of the respondents confirmed the question about the opportunity of supplementing the fruits and vegetable consumption, while 20.77% confirmed that fruit and vegetable consumption is equally useful to health as meat consumption (37). In regards to the fruits and vegetable consumption, the main motivation for the increase in their consumption in order to improve their health represents their characteristic regarding the protection of the immune system, the easiness of their processing by the metabolic system, and the increase in food safety and health (52.82% of the respondents). This suggests that the target group felt certain about the desirable health effects attributed to fruits and vegetable consumption. The study also shows that only 25.24% admit that an increase in fruits and vegetable consumption would be motivated by their qualitative increase (bio products), while 12.2% consider the price as an impediment to fruits and vegetable consumption (these aspects are shown in Figure 4). This fact suggests that consumers are paying more attention to the benefits of their consumption and want to have a greater control over food, being concerned about the processing of the food they consume (41).

**FIGURE 5:**
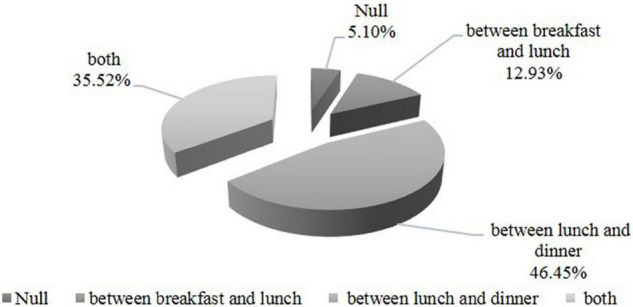
The consumers’ options regarding the consumption of snacks and the moment of having it.

In regards to the supplementary meat consumption that helps increase the health of the population, the majority admit that they would eat more meat if it were less noxious, while only 7% of those who opted for the supplementary meat consumption consider that the major impediment in meat consumption is given by its price. The majority of respondents (68.85%) admitted that the main meals are sometimes followed by a dessert, while 23.13% of the respondents confirm the habit of completing the main meals with a dessert. Only 8% of the respondents do not usually complete their daily meals with other supplementary caloric intakes. One can notice that the majority of the respondents use the afternoon time frame between lunch and dinner to have a dessert (46.45%), while only 12.93% have dessert between breakfast and lunch. At the same time, 5.1% of the respondents failed to offer a valid answer to this question whereas 35.52% admitted that they did not have food preferences regarding the specific moment of having the dessert between meals, as shown in Figure 5.

In regards to the consumption of organic products (42, 43), the questionnaire indicated that a percentage of 61% of the population prefer the consumption of organic products, while only 39% of the population prefer the traditional products. These results are in line with previous research, in which a “self-reported scientific education was characteristic of greater consumption of organic produce” (44, 45). Therefore, scientific education can influence the likelihood of green purchasing. The consistency of the respondents regarding the good practice of food intake is proven by a rare visit to restaurants serving fast food (i.e., one time a month or more rarely for 63.39% of respondents and never for 7.1% of the respondents). In regards to serving meals in fast-food type of restaurants, the study shows that only 29.51% prefer this place for having a meal. Out of these, 2.19% use this place daily and at least 1 time a week (27.32%). In regards to having a meal in specialized places (i.e., restaurants, cafeterias, etc.), the majority of the respondents (60.47%) declare that they seldom use the specialized restaurants or very seldom (i.e., 51.37% occasionally, 9.11% never). The individuals who frequently use the cafeterias or restaurants do not reach more than 2% of the total of the respondents’ options. The rest of the options (37.52%) are for those who mostly go to specialized restaurants.

### Decision-Makers in Purchasing Food

The third section of the questionnaire focuses on the types of decisional factors, the purchase frequency, food preferences, the information regarding the nutritional value and other elements included on the label, and the impact of the commercial aspect of the product, among others.

As has been shown previously in other studies (46), in terms of the decisional factors, our study reveals that the majority of consumers consider that smell, taste, and flavors influence the purchase decision (50.09%). Second comes the consumers’ evaluation of the nutritional intake, where 33.5% of the respondents failed to define their consumption decisions depending on the organoleptic features of the product. The act of purchase takes place quite frequently, one time or two times a week (55.19%) or daily (15.66%), while about 20% of the respondents admit the fact that they shop one time every 10–15 days.

Our study confirms the results of previous studies conducted by other researchers (47–50) according to which the social/environmental (social class, culture, reference groups, and peer groups), personal (perception, personality, attitude, and motivation), individual (gender, age, income, and education), and marketing mix factors (product, pricing, promotion, and place) are the main factors that influence consumers’ behavior in making decisions related to food consumption. Results of surveys concerning consumers’ preference for local food and consumers’ environmental and ethical consciousness demonstrate that 46.8% of the respondents are willing to pay for local food out of their wish to support the local economy, patronage of farmers’ markets, and consumer ties to agriculture (51). The study also shows that 42.62% of the respondents appreciate and have positive attitudes toward the environmental and social aspects of food, but as stated in other studies (52), these attitudes are not always reflected in their behavior.

The buyers’ options regarding the food consumption depending on the culinary preferences, analyzed in balance with the options regarding maintaining health through correct nutrition, emphasize that the majority of the consumers are aware of the direct impact of nutrition on health (65.03%), while only 30% of the consumers choose the food tradition over the prejudice of health.

Structuring the diet depending on different qualitative or quantitative characteristics (adapting the diet to the area characteristic, maintaining a diet according to the food traditions), cognition regarding the objective features and attributes of the consumed products analyzed in relationship with the financial limitation in the financial structure of a varied, suitable and diversified menu emphasize that, in 38.8% of the cases, the financial availabilities dictate the diet that emerges as the main factor of influencing the diversity of the food menus, while the food traditions are situated on the second place, being 17.67% of the consumers’ preferences.

The degree of adaptability to specific requirements and the destination supply is a low factor, amounting to 10% of consumer options. The results of the study show that young and educated consumers are quite confused regarding their active role as influencing factors in the food market due to the fact that, although they have access to information, they base their decisions on food consumption mainly based on recommendations. If the diet is influenced by income, as we have shown, the purchase decision is influenced preponderantly by the friends’ or the family’s recommendations (53). In other words, there is a little degree of trust regarding the information from media or the traditional promoting channels (fairs and exhibitions), with the consumer being directly interested in a product already tested by trustful persons or that at least has a qualitatively attractive commercial look.

This observation is significant regarding the objective of the research because it proves the failure of the classical measures of promoting and emphasizes an adaptive consumers’ behavior depending on the level of trust obtained by testing or self-testing the purchased food products. This aspect only confirms that there is a high degree of information asymmetry among the questioned population. An interesting and extensive debate has been made by many scholars to identify ways to reduce asymmetric information between corporate sustainability efforts and consumers by developing different types of eco-labels (54). The notoriety of the information on the label is partially recognized by the consuming population, so from the questionnaire achieved, the results show that the majority of the responders use labeled products usually in commerce (33.52%), while in the second place is the organic products (23.68%), and 20.04% of the responders declare that they buy products irrespective of their way of being labeled.

### Information Asymmetry

The fourth section deals with the information asymmetry and contains 11 questions that were meant to determine the consumers’ profile based on the consumption typology, the products of protected origin, the organic products, the genetically modified products/nongenetically modified products linked to the financial allocation in the daily basket of goods of the net nutritional intake. This section brings valuable information about the food consumption habit of a controlled and protected origin (that can be rarely observed as part of the population’s consumption – 63.21%, or even unknown by 24.5% of the respondents). These results lead to the idea that there is a high degree of informational asymmetry among young consumers regarding the consumption of food of a controlled and protected origin. We believe that this aspect can only be improved by food policies of national and international bodies that will contribute to greater visibility and knowledge of these products among young consumers. Also, these policies should be doubled by the efforts of marketers oriented toward familiarizing consumers with these types of products.

Our study reveals that 66.67% of the respondents admit that they use organic products from time to time, while only 28.96% of the respondents admit that they frequently use organic products. This trend is also maintained in the case of those products that are rich in nutrients or do not contain genetically modified organisms whose cost is higher. The results of our study show that young and educated consumers have a need to consume food that is beneficial to their health, but their financial possibilities are limited, confirming hypothesis 2. The products with inelastic demand such as coffee, chocolate, etc. maintain their notoriety on the market as they are consumed on a regular basis by 53.37% of the respondents, while a percentage of 43.72 as a result of realizing the harmful effects they have upon health limit their consumption. GMOs are not part of the consumption preferences of the consumers, 65.21% of the respondents admit that they use them sparingly or very rarely, while 28.42% admit that they never use them. This suggests that most consumers know very little about agricultural technologies (55). The analysis of literature data indicates that simply “educating” consumers with regard to scientific information about these technologies is unlikely to significantly influence consumer perception, due to consumers’ previous behavioral beliefs and biases (56, 57). The financial structure of the cost of the consumption per daily meal varies between 10 and 20 lei^1^ on average depending on the type of the meal as follows:

1. Breakfast has an average financial allocation of 5–15 lei (73.22%);
2. Lunch has an average financial allocation of between 10 and 20 lei with special attention to the 15–20 lei (34.24%) segment, while, for the 10–15 lei (33.7%) segment, the representation of the options is more reduced due to the inferior nutritional food intake and the small cost difference between the two segments (i.e., the equivalent value of a complete meal at the faculty cafeteria is of about 15 lei). The more expensive (i.e., over 20 lei) qualitative nutritional menus are rarely used by the respondents (24.23%).
3. Dinner has a financial allocation budgeted between 10 and 15 lei, which ensures on average a sufficient nutritive intake for the consumer;
4. The financial allocations for snacks in between meals do not surpass 10 lei.

The aforementioned confirm that the consumption behavior of the educated young people, their decision to purchase, the structure of the budget allocated to food consumption, and the daily component of the meals are influenced by the informational asymmetry, thus validating hypotheses nos. 3 and 5.

The total value of the food basket does not surpass 55% of the income per household according to 93.44% of the respondents. In order to demonstrate the hypotheses of the research, three sections of interest regarding information asymmetry as a disruptive factor in consumers’ behavior have been established based on the information shown in Table 6. The three sections are defined as follows:

**TABLE 6:** The sections of interest regarding information asymmetry in relationship with the hypotheses and the mapping of the questions.

Sections	Split criteria Q5 (family working members)	Hypotheses				Questions													
1. Characteristics	Functional	H1	H3			Q6	Q8	Q9	Q10	Q11	Q12	Q13	Q14	Q15	Q16	Q23	Q27	Q33	Q34
	Economic	H1	H2	H4	H5	Q3	Q2	Q4	Q21	Q22	Q27	Q36	Q37	Q38	Q39	Q40			
	Technical	H3				Q29	Q30	Q31	Q32	Q35									
	Space	H4				Q7	Q27												
2. Attributes	Perceived quality	H1	H4			Q18	Q19												
	Expected quality	H1	H2			Q18	Q19	Q23	Q25	29	Q30	Q31	Q32	Q35					
3. Causality	Consumer decision	H1	H4			Q2	Q3	Q5	Q17	Q20	Q22	Q24	Q26	Q28	Q29	Q30	Q31	Q32	Q35
	Need for consumption	H1	H2			Q6	Q24	Q26	Q33	Q38	Q39	Q40							

In order to develop the pattern of informational asymmetry, as a disturbing factor of consumer behavior in the food market, we proceeded to assign the survey questions to sections of interest regarding information asymmetry in relationship with the hypotheses and the mapping of the questions. The model of information asymmetry was defined based on the statistical program Gretl, version 2019, as follows:


$$A\,I=S_{1}\,⋂S_{2}\,⋂S_{3}=$$

(1) ∑j⁢14∏i⁢15Hi1⋂∑j⁢12∏I⁢14HI2⋂∑j⁢12∏I⁢14HI3=(∑i=140max(α(Qi))Si

In order to estimate the parameters of the model, the criterion of segregation of the information that was processed on the basis of the questionnaire comprised by question no. 5 was used (i.e., the number of members in the household that contribute to the family income). The levels of the maximum value for each question in the questionnaire have been calculated. They were obtained on a scale of k options (*k* = $\bar{1,8}$ where 8 represents the maximum number of options of a question *k* > 1). Thus, the values that have resulted for each question have been compared based on the criterion of segmentation of the questionnaire (Q5). Thus, four models of behavior were obtained based on the completive (S1), attributive (S2), and causal (S3) information asymmetry.

Unlike the specialized literature where the information asymmetry is analyzed mainly from the informational volume point of view (more concretely from the perspective of the quantity and detail of the information), this study promotes for the first time the concept of attributive, causal, complementary and residual asymmetry. The reasoning behind the promotion of these concepts is strictly based on the quality of the information that is confined to this type of information flow. We mention this aspect because the normative treatment of the qualitative characteristics of the information can be found within the International Accounting Standards Board framework. According to this reference, attributes such as relevance, opportunity, verifiability, comparability, and intangibility decisively contribute to the foundation of consumer decisions. Based on this consideration, a future research direction could consist of the transposition and correlation of these qualitative characteristics on the model of the information requested by the consumers of food products and of course the determination of their weights on each characteristic that influences the consumption behavior.

The values that resulted from the use of the model are presented synthetically in Table 7. As can be seen from the table below, the completive asymmetry through its functional and economic sides in relationship with the attributive asymmetry assigned to the desired quality of the product has the biggest impact on the consumers’ changes in behavior. This thing is correlated with the fact that, within the three sections generating the asymmetry, the work hypotheses are the most visible ones (*i* = 5). The next element of information asymmetry refers to the causal asymmetry (i.e., the aspects regarding the consumers’ decision) that in the light of subjectivity and behavior may influence decisively the consumption decision. Thus, in order to cancel it or double it sometimes, it is indirectly proportional to the need for consumption. A residual information asymmetry is generated in section 1. It is suggested by the technical features of the products on the market as well as by the attributive asymmetry on the perceived quality of the products. Thus being considered in relationship with one another, the expected quality generates the information asymmetry which is much higher than the perceived quality. On the basis of the studies presented in the study and their results, it could also be said that the migration of young and educated consumers and traditional-zonal consumption (complementary asymmetry) are elements with resistance to informational asymmetry, a fact which validates the study hypothesis 4.

**TABLE 7:** The estimation of the features of the information asymmetry model is based on the Q5 segmentation criterion.

Sections	Split criteria Q5					All	Working members – 1	Working members – 2	Working members – 3	Working members – +3
Sections 1 – characteristics	Functional	H1	H3			44.00	45.00	42.00	46.00	42.00
Sections 1 – characteristics	Economic	H1	H2	H4	H5	25.00	23.00	26.00	24.00	27.00
Sections 1 – characteristics	Technical	H3				13.00	13.00	13.00	13.00	16.00
Sections 1 – characteristics	Space	H4				3.00	2.00	3.00	3.00	3.00
Section 2 – attributes	Perceived quality	H1	H4			3.00	3.00	3.00	6.00	3.00
Section 2 – attributes	Expected quality	H1	H2			19.00	20.00	19.00	22.00	23.00
Section 3 – causality	Consumer decision	H1	H4			36.00	33.00	36.00	34.00	41.00
Section 3 – causality	Need for consumption	H1	H2			19.00	18.00	20.00	19.00	19.00

*Q, questions; H, hypotheses.*

In the elaboration of the table above, we had as a starting point the items used by Marinelli (20). Starting from this correlation, the results of the study revealed that the attributes that the information provided by the producer to the consumer regarding the quality of the products must fulfill, have a direct, positive, and lasting impact on reducing the information asymmetry, thus determining a certain typology of consumer behavior. Another aspect that the study highlighted was that the target group showed an informational asymmetry given by the level of education and financial resources allocated to global consumption per household, thus confirming hypothesis 1. These findings are in line with other international studies (58) on information asymmetry in the food market, which show that the more a consumer knows about food, the more likely they are to look for the information on the product packaging. Nestorowicz (58) argues that the reduction of information asymmetry is achieved when consumers seek information, thus leading to an equalization of the level of knowledge between the consumer and the producer.

The analysis of data allowed us to identify the main factors that determine the consumers’ behavior and perception toward the quality food products and the level of consumption regarding certain quality product categories, as well as factors of constraint in following and observing a healthy diet.

The results of the survey led both to the identification of a quantification model of the information asymmetry that manifests itself within the relationship between the producer and the consumer and to the identification of a typology of informational asymmetry which manifests itself differently depending on the features of the food products.

## Conclusion

The present study suggested the quantification of the impact of the information asymmetry on the consumers’ behavior which, in its turn, has a direct influence on the food policies applied by the national and international bodies. The research helps to better understand the attitudes of young and educated consumers toward the individual values and beliefs they use in food choices.

The priority research areas concerned the informational asymmetry through the relation between the producer and the consumer, the typology of the consumption, the influence of the food policies on the consumer’s behavior, the determining factors in the decision-making process of food consumption, etc. In this sense, the information asymmetry has been studied based on a questionnaire that was applied to a number of over 500 respondents whose level of education is superior and who possess relevant knowledge regarding the impact of consumption typology on health. Considering that the young and educated population represents an important segment with an increasing purchasing power, which is also of interest to the regulatory bodies in order to develop food policies, we considered their selection as a target group a real interest for the study undertaken.

The data that were obtained as the result of the questionnaire have been adjusted on the basis of the model of information asymmetry designed by the authors. It is an original model based on mixing the work hypotheses with the observations given as a result of the study of the questionnaire and the segmentation that was made according to the number of members that contribute to the family income. The model is a cumulative type that pointed out the impact of information asymmetry on the consumers’ behavior depending on the completive (S1), attributive (S2), and causal (S3) sections.

However, our finding suggests that educated young people have different styles of eating behavior, inspired or based on different principles that lead to greater or lower availability in terms of supporting or practicing “sustainable” eating behaviors and in line with food policies. The study also emphasizes the fact that a significant part of the sample is represented by people who are aware of or who pay attention to their own health and sustainable consumption but also by people who are concerned or more selective regarding the information on the origin and the way of obtaining food. Furthermore, the study reveals that a high share of the population surveyed is willing to change their choice or consumption behavior if this choice is of an ecological and sustainable nature.

Our finding suggests that, if the food model is influenced by the level of income, the purchase decision is influenced by the information obtained from close friends or relatives. The results also showed that there is a low degree of confidence in the information in the media or in the traditional promotion channels. These findings are significant in terms of the research objective as it proves the failure of the traditional promotion measures and highlights an adaptive behavior of consumers depending on the level of confidence gained through testing or self-testing of the purchased food products.

The model developed for the information asymmetry is of real use in influencing or even determining the consumption behavior of the analyzed target group. Our study highlighted the fact that there are three determinants (causal nature, the quality characteristics of the information provided by the producers, and the completeness of this information), which can reduce, increase or keep the information asymmetry created between the producer and the consumer. The demonstration that the information asymmetry is at the intersection of the three segments mentioned above, confers utility and importance to the model. We support this assertion because any producer interested in satisfying the demands of consumers as much as possible will always be able to monitor to what extent he can minimize the costs associated with reducing information asymmetry (in order to optimize communication with his consumers). This fact can be made possible by segregating these quality attributes and focusing the attention, especially on the segment considered to be the most important by consumers. From the consumer perspective, our model of informational asymmetry can represent decisive informational support in the choice of the producer.

The model can be used based on the factors defining the present food policy and the projections for establishing the possible necessary changes that are generated by the information asymmetry comprised by the segments of consumers defined by the incident criteria such as the food culture, the financial value, a consolidated family group, the update information based on the process of continuous training, and the change in the need for consumption under the impact of the modification of the offer of goods and services as a result of technologic adjustments. The results can be also useful to the marketers in order to substantiate the marketing policies, the bodies with a role in consumer education in order to help them to follow healthy nutrition.

Based on the informational asymmetry model offered by the authors and the results obtained from the target group questionnaires, the regulatory and control bodies together with the research institutes in the health, social, educational fields, etc., can elaborate a manual/guide for the orientation of the consumption behavior, especially in the conditions of the COVID-19 pandemic, when the decisions were based even more on the consumers’ income, which certainly influenced the quality of the food provided. At the same time, they can develop some quality nutrition policies that contribute to maintaining the health of the population. These measures should encourage the reduction of the consumption of unhealthy foods, with implications for the sustainability of resources and the protection of the environment.

The limitations of the study include the use of the maximum criterion in the selection of the median options for the respondents that were segmented according to the criteria, as well as the restriction of the model only when applying the principle of the number of individuals that contribute at the family income. For future research, we suggest the inclusion of further segmentation criteria that will emphasize the new types of information asymmetry in relationship with their completive (S1), attributive (S2), and causal (S3) values. We believe that our study can be a scientific step in improving the specialized literature in this field.

## Data Availability Statement

The raw data supporting the conclusions of this article will be made available by the authors, without undue reservation.

## Ethics Statement

Ethical review and approval was not required for the study on human participants in accordance with the local legislation and institutional requirements. The patients/participants provided their written informed consent to participate in this study.

## Author Contributions

MS, VG, M-SC, and S-MB: conceptualization. MS, VG, and M-SC: methodology and writing—original draft preparation. MS, S-MB, and CC: data curation. M-SC, S-MB, and CC: formal analysis, investigation, and visualization. MS, CC, and VG: supervision and validation. All authors contributed to the article and approved the submitted version.

## Conflict of Interest

The authors declare that the research was conducted in the absence of any commercial or financial relationships that could be construed as a potential conflict of interest.

## Publisher’s Note

All claims expressed in this article are solely those of the authors and do not necessarily represent those of their affiliated organizations, or those of the publisher, the editors and the reviewers. Any product that may be evaluated in this article, or claim that may be made by its manufacturer, is not guaranteed or endorsed by the publisher.

## Footnotes

1 All the values are denominated in the national currency of Romania, the LEU; 1 euro = 4.7336 lei based on the exchange rate of the Romanian National Bank on 31-07-2019.

## References

[1] Kafel P. Food quality products in EU countries. *Presented at 7th International Quality Conference, Center for Quality, Faculty of Engineering, University of Kragujevac.* Kragujevac: University of Kragujevac (2013). p. 273–8.

[2] Yuan X Li C Zhao K Xu X. The changing patterns of consumers’ behavior in China: a comparison during and after the COVID-19 pandemic. *Int J Environ Res Public Health.* (2021) 18:2447. 10.3390/ijerph18052447 33801491 PMC7967584

[3] Yazdanparast A Alhenawi Y. Impact of COVID-19 pandemic on household financial decisions: a consumer vulnerability perspective. *J Consum Behav.* (2022) 2022:1–22. 10.1002/cb.2038 PMC9083242 37519436

[4] World Health Organization Regional Office. *European Food and Nutrition Action Plan.* (2018). Available online at: www.euro.who.int/__./64wd14e_FoodNutAP_140426.pdf (accessed March 1, 2019).

[5] Akerlof GA. The market for “Lemons”: quality uncertainty and the market mechanism. *Q J Econ.* (1970) 84:488–500.

[6] Spence M. Job market signaling. In: Diamond P Rothschild M editors. *Uncertainty in Economics.* Cambridge, MA: Academic Press (1978). p. 281–306. 10.1016/B978-0-12-214850-7.50025-5

[7] Stiglitz JE Weiss A. Asymmetric information in credit markets and its implications for macro-economics. *Oxf Econ Pap.* (1992) 44:694–724.

[8] Nestorowicz R. The asymmetry of information on food market and consumers’ preferences-some aspects. *Zesz Nauk Szk Gł Gospod Wiej W Warszawie Polityki Eur Finanse Mark.* (2013) 10:506–13.

[9] Min S Cheng X Zhang X-H. Impacts of the COVID-19 pandemic on consumers’ food safety knowledge and behavior in China. *J Integr Agric.* (2020) 19:2926–36. 10.1016/S2095-3119(20)63388-3 35755618 PMC9215339

[10] Grunert KG. Food quality and safety: consumer perception and demand. *Eur Rev Agric Econ.* (2005) 32:369–91. 10.1093/eurrag/jbi011

[11] Galati A Tulone A Moavero P Crescimanno M. Consumer interest in information regarding novel food technologies in italy: the case of irradiated foods. *Food Res Int.* (2019) 119:291–6. 10.1016/j.foodres.2019.01.065 30884659

[12] Beck AM Balknäs UN Fürst P Hasunen K Jones L Keller U Food and nutritional care in hospitals: how to prevent malnutrition–report and guidelines from the Council of Europe. Council of Europe (the committee of experts on nutrition, food safety and consumer health of the partial agreement in the social and public health field). *Clin Nutr.* (2001) 20:455–60. 10.1054/clnu.2001.0494 11534942

[13] Stigler GJ. The economics of information. *J Polit Econ.* (1961) 69:213–25. 10.1086/258464

[14] Cheung WM Chung R Fung S. The effects of stock liquidity on firm value and corporate governance: endogeneity and the REIT experiment. *J Corp Finan.* (2015) 35:211–31. 10.1016/j.jcorpfin.2015.09.001

[15] Nelson P. Information and consumer behavior. *J Polit Econ.* (1970) 78:311–29. 10.1086/259630

[16] Darby MR Karni E. Free competition and the optimal amount of fraud. *J Law Econ.* (1973) 16:67–88. 10.1086/466756

[17] Mazzocchi M Traill W Shogren J. *Fat Economics: Nutrition, Health, and Economic Policy.* Oxford: Oxford University Press (2009).

[18] Miller M. Big data, information asymmetry, and food supply chain management for resilience. *J Agric Food Syst Community Dev.* (2021) 11:171–82. 10.5304/jafscd.2021.111.017

[19] Ramírez Ó Charry A Díaz MF Enciso K Burkart S. The effects of COVID-19 on beef consumer preferences and beliefs in colombia: a logit model approach. *Front Sustain Food Syst.* (2021) 5:725875. 10.3389/fsufs.2021.725875

[20] Marinelli N. Asimmetrie informative e sicurezza alimentare nei diritti del consumatore e nella competitività dei sistemi produttivi. *Ital J Agron.* (2010) 4:13–21.

[21] Artene A Domil A. Sustainable development in agricultural collective companies by identifying and following environmental costs. *Lucr ?tiin?ifice Univ ?tiin?e Agric ?i Med Vet Banat Timisoara Ser Manag Agric.* (2012) 14:223–8.

[22] Fidrmuc J Schreiber P Siddiqui M. Information asymmetry, relationship banking and financing costs of SME’s. *SSRN Electron J.* (2015) 2015:2565350. 10.2139/ssrn.2565350

[23] Jiménez JGM Santiago AR López-Herrera F. Measuring the asymmetry level around quarterly reports in the Dow Jones, Nasdaq, and Standard & Poor’s: before and during the COVID-19 pandemic. *Invest Anal J.* (2021) 50:50–9. 10.1080/10293523.2021.1876826

[24] Nestorowicz R. The information asymmetry and the social responsibility on the food market. *Int J Arts Sci.* (2014) 7:59.

[25] Iyengar SS Lepper MR. When choice is demotivating: can one desire too much of a good thing? *J Pers Soc Psychol.* (2000) 79:995–1006. 10.1037/0022-3514.79.6.995 11138768

[26] Arunachalam B Henneberry SR Lusk JL Norwood FB. An Empirical Investigation into the Excessive-Choice Effect. *Am J Agric Econ.* (2009) 91:810–25. 10.1111/j.1467-8276.2009.01260.x

[27] McCluskey JJ Loureiro ML. Consumer preferences and willingness to pay for food labeling: a discussion of empirical studies. *J Food Distrib Res.* (2003) 34:95–102.

[28] McCluskey JJ Grimsrud KM Ouchi H Wahl TI. Consumer response to genetically modified food products in Japan. *Agric Resour Econ Rev.* (2003) 32:222–31. 10.1017/S1068280500005992

[29] Muresan IC Harun R Arion FH Brata AM Chereches IA Chiciudean GO Consumers’ attitude towards sustainable food consumption during the COVID-19 pandemic in Romania. *Agric.* (2021) 11:1050. 10.3390/agriculture11111050

[30] Di Palma A. *Factors Influencing Consumers’ Online Purchase Behaviour of SkinCare*. Masters thesis. London: University of East London Royal Docks BusinessSchool (2015).

[31] Le HQ Nguyen TM. Behaviors in the market for safe vegetables under information asymmetry: modeling approach. *Eurasian Econ Rev.* (2018) 8:381–92. 10.1007/s40822-018-0093-5

[32] Rezitis AN Tsionas M. Modeling asymmetric price transmission in the european food market. *Econ Model.* (2019) 76:216–30. 10.1016/j.econmod.2018.08.004

[33] Palma MA Collart AJ Chammoun CJ. Information asymmetry in consumer perceptions of quality-differentiated food products. *J Consum Aff.* (2015) 49:596–612. 10.1111/joca.12053

[34] Grant IC Waite K. “Following the Yellow Brick Road”–young adults’ experiences of the information super-highway. *Qual Mark Res Int J.* (2003) 6:48–57.

[35] Grønhøj A. The consumer competence of young adults: a study of newly formed households. *Qual Mark Res Int J.* (2007) 10:243–64.

[36] Jakubowska D Radzymińska M. Health and environmental attitudes and values in food choices: a comparative study for Poland and Czech Republic. *Oeconom Copernic.* (2019) 10:433–52.

[37] Chiciudean D Arion F Mureşan I Chiciudean G. Consumer behaviour of local meat and meat products. *ProEnvironment.* (2018) 11:63–9.

[38] Chiciudean GO Harun R Ilea M Chiciudean DI Arion FH Ilies G Organic food consumers and purchase intention: a case study in Romania. *Agronomy.* (2019) 9:145. 10.3390/agronomy9030145

[39] Wang E An N Gao Z Kiprop E Geng X. Consumer food stockpiling behavior and willingness to pay for food reserves in COVID-19. *Food Secur.* (2020) 12:739–47. 10.1007/s12571-020-01092-1 32837661 PMC7406878

[40] USDA. *The Food Guide Pyramid.* (2009). Available online at: https://www.cnpp.usda.gov/sites/default/files/archived_projects/FGPPamphlet.pdf (accessed March 29, 2021).

[41] Lusk JL McCluskey J. Understanding the impacts of food consumer choice and food policy outcomes. *Appl Econ Perspect Policy.* (2018) 40:5–21. 10.1093/aepp/ppx054

[42] McCluskey JJ. A game theoretic approach to organic foods: an analysis of asymmetric information and policy. *Agric Resour Econ Rev.* (2000) 29:1–9. 10.1017/S1068280500001386

[43] Oroian C Safirescu C Harun R Chiciudean G Arion F Muresan I Consumers’ attitudes towards organic products and sustainable development: a case study of Romania. *Sustain.* (2017) 9:1559. 10.3390/su9091559

[44] Lockie S Lyons K Lawrence G Mummery K. Eating ‘Green’: motivations behind organic food consumption in Australia. *Soc Rural.* (2002) 42:23–40. 10.1111/1467-9523.00200

[45] Nunez GH Kovaleski AP Darnell RL. Formal education can affect students’ perception of organic produce. *Hort Technol.* (2014) 24:64–70. 10.21273/HORTTECH.24.1.64 35581909

[46] Chiciudean D Funar S Arion F Chirla G Man A. The factors of influence over the consumer buying behaviour for organic food. *Bull Univ Agric Sci Vet Med Cluj Napoca Hortic.* (2012) 69:68–71.

[47] Sandu MC. Important elements in consumer’s decision-making process. *Calitatea Vieţii.* (2014) 25:365–73.

[48] Dumitru M. Important elements in consumer’s decision-making process. *Proc Econ Finance.* (2015) 22:780–6. 10.1016/S2212-5671(15)00306-8

[49] Hamam M Di Vita G Zanchini R Spina D Raimondo M Pilato M Consumers’ attitudes and purchase intention for a vitamin-enriched extra virgin olive oil. *Nutrients.* (2022) 14:1658. 10.3390/nu14081658 35458217 PMC9027912

[50] Di Vita G Zanchini R Spina D Maesano G La Via G D’Amico M. Exploring purchasing determinants for a low fat content salami: are consumers willing to pay for an additional premium? *Front Sustain Food Syst.* (2022) 6:794533. 10.3389/fsufs.2022.794533

[51] Carpio CE Isengildina-Massa O. Consumer willingness to pay for locally grown products: the case of South Carolina. *Agribus Int J.* (2009) 25:412–26. 10.1002/agr.20210

[52] Ghvanidze S Velikova N Dodd TH Oldewage-Theron W. Consumers’ environmental and ethical consciousness and the use of the related food products information: the role of perceived consumer effectiveness. *Appetite.* (2016) 107:311–22. 10.1016/j.appet.2016.08.097 27554182

[53] Kakiza C. *Factors Affecting Purchasing Decisions of the Consumers: A Case of Kinondoni District (Dar es salaam).* Ph. D. Thesis. Tanzania: Mzumbe University (2015).

[54] Nikolaou IE Kazantzidis L. A sustainable consumption index/label to reduce information asymmetry among consumers and producers. *Sustain Prod Consum.* (2016) 6:51–61. 10.1016/j.spc.2016.01.001

[55] Huffman WE Rousu M Shogren JF Tegene A. The effects of prior beliefs and learning on consumers’ acceptance of genetically modified foods. *J Econ Behav Organ.* (2007) 63:193–206. 10.1016/j.jebo.2005.04.019

[56] Kahan DM Braman D Slovic P Gastil J Cohen G. Cultural cognition of the risks and benefits of nanotechnology. *Nat Nanotechnol.* (2009) 4:87–90. 10.1038/nnano.2008.341 19197308

[57] McFadden BR Lusk JL. What consumers don’t know about genetically modified food, and how that affects beliefs. *FASEB J.* (2016) 30:3091–6. 10.1096/fj.201600598 27199295

[58] Nestorowicz R. Information asymmetry and the effectiveness of marketing communications on health-oriented food market. In: Bilgin MH Danis H editors. *Entrepreneurship, Business and Economics.* (Vol. 1), Cham: Springer International Publishing (2016). p. 525–34. 10.1007/978-3-319-27570-3_39

[59] Zhai Y Han G. The effect of the inspection information sharing policy on quality-oriented food production in online commerce. *Manag Decis Econ.* (2022) 43:84–96. 10.1002/mde.3360

[60] Bronnmann J Stoeven M Quaas M Asche F. Measuring motivations for choosing eco-labeled seafood: environmental concerns and warm glow. *Land Econ.* (2021) 97:641–54. 10.3368/wple.97.3.101119-0147R

[61] Brach S Walsh G Shaw D. Sustainable consumption and third-party certification labels: consumers’ perceptions and reactions. *Eur Manag J.* (2018) 36:254–65. 10.1016/j.emj.2017.03.005

[62] Plank A Teichmann K. A facts panel on corporate social and environmental behavior: decreasing information asymmetries between producers and consumers through product labeling. *J Clean Prod.* (2018) 177:868–77. 10.1016/j.jclepro.2017.12.195

